# Supplementary material for: Moisture-triggered fast crystallization enables efficient and stable perovskite solar cells
Source: Nat Commun. 2022 Aug 19;13:4891. doi: 10.1038/s41467-022-32482-y (PMC9391447; doi:10.1038/s41467-022-32482-y)
Supplement: Supplementary file 1 — Supplementary Information [file 41467_2022_32482_MOESM1_ESM.pdf]

Supplementary Information

Moisture-triggered Fast Crystallization Enables Efficient and Stable Perovskite Solar Cells

Kaikai Liu[1,†], Yujie Luo[1,†], Yongbin Jin[1], Tianxiao Liu[2], Yuming Liang[1], Liu Yang[1], Peiquan Song[1], Zhiyong Liu[2], Chengbo Tian[1], Liqiang Xie[1,*] & Zhanhua Wei[1,*]

[1] Xiamen Key Laboratory of Optoelectronic Materials and Advanced Manufacturing, Institute of Luminescent Materials and Information Displays, College of Materials Science and Engineering, Huaqiao University, Xiamen 361021, P.R. China

[2] Henan Key Laboratory of Photovoltaic Materials, School of Physics, Henan Normal University, Xinxiang 453007, P.R. China

[†] These authors contributed equally to this work.

E-mails: lqxie@hqu.edu.cn; weizhanhua@hqu.edu.cn

This Supplementary Information includes:

Supplementary Figures 1 to 24

Supplementary Note 1 and Supplementary Figure 25

Supplementary Tables 1 to 3

Supplementary Methods

References

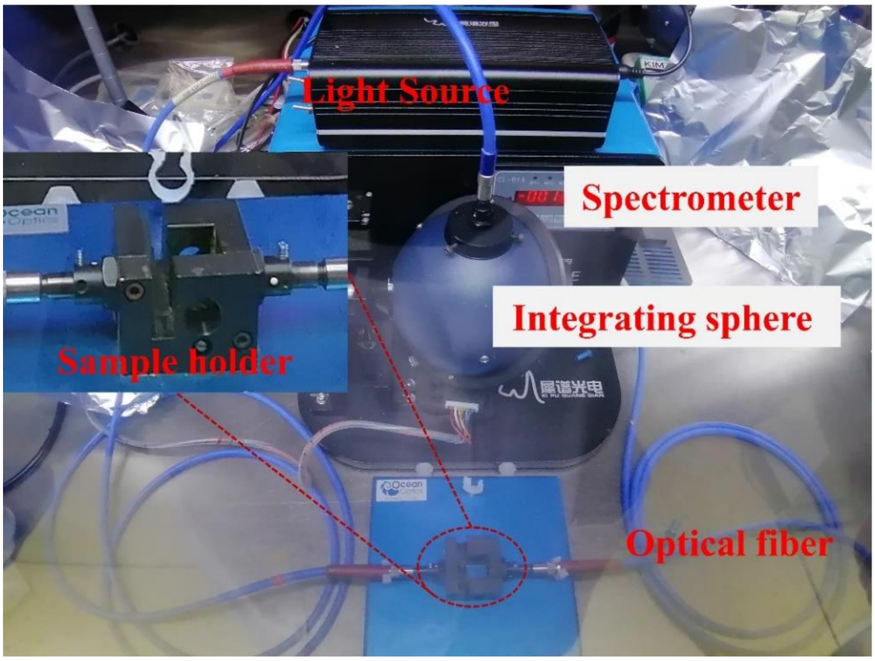

Supplementary Figure 1 | Photograph of the setup for measuring the UV-vis spectra and PL spectra in N_2. The setup is placed in an N_2-filled glove box. It contains a light source, a sample holder, two optical fibers, an integrating sphere, and a spectrometer.

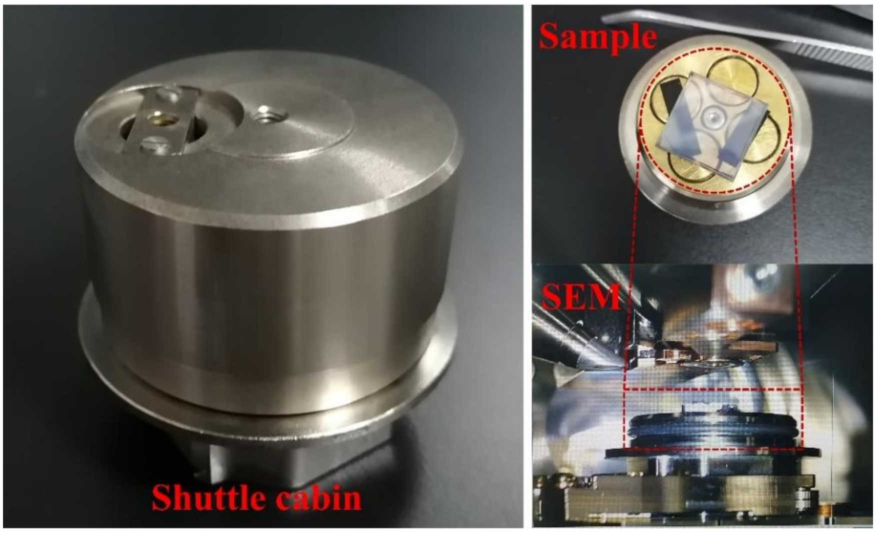

Supplementary Figure 2 | Photographs of the protective SEM measurement setup. The protective shuttle cabin for SEM measurement (left), the sample fixed in the shuttle cabin (upper right), and measuring the sample in the SEM equipment (bottom right).

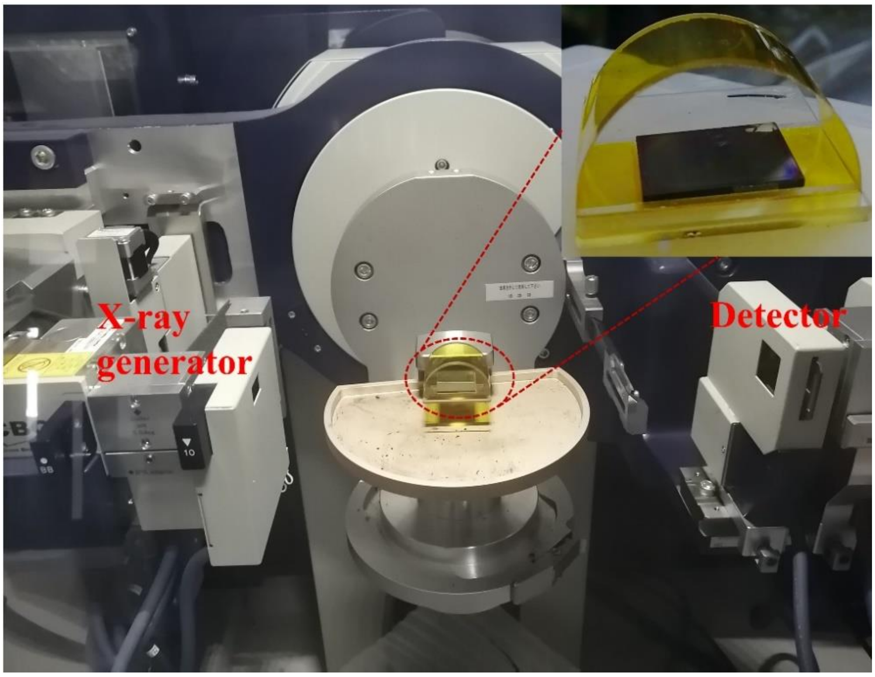

Supplementary Figure 3 | Protective XRD characterization. Photograph of the XRD characterization equipment with the protection cabin. Inset in the upper right corner shows the sample fixed in the protective cabin in N_2.

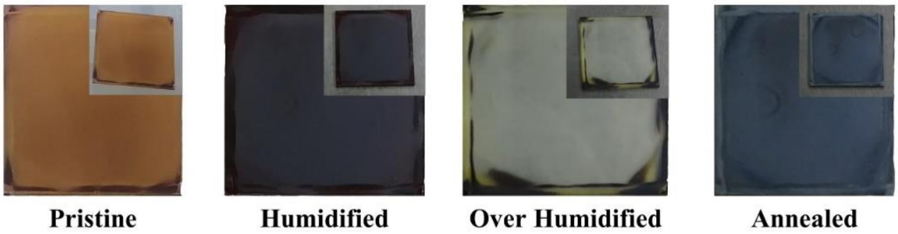

Supplementary Figure 4 | Colour change of the same perovskite film in different stages. Photographs of the same wet intermediate perovskite film in stages of Pristine, Humidified, Over Humidified, and Annealed.

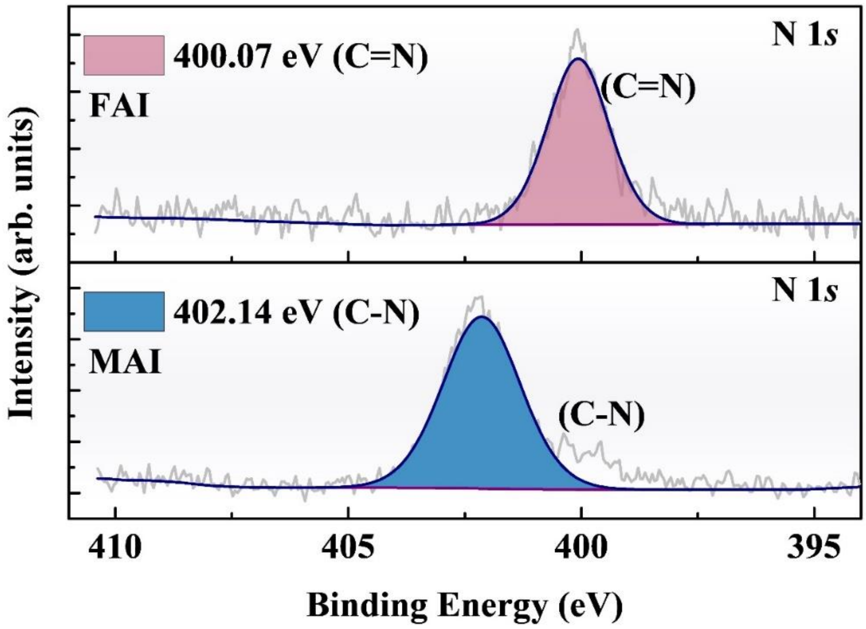

Supplementary Figure 5 | XPS spectra of the FAI and MAI samples. From the N 1*s* signal, it can be confirmed that the C=N bond of FAI locates at about 400.07 eV and the C-N bond of MAI locates at about 402.14 eV.

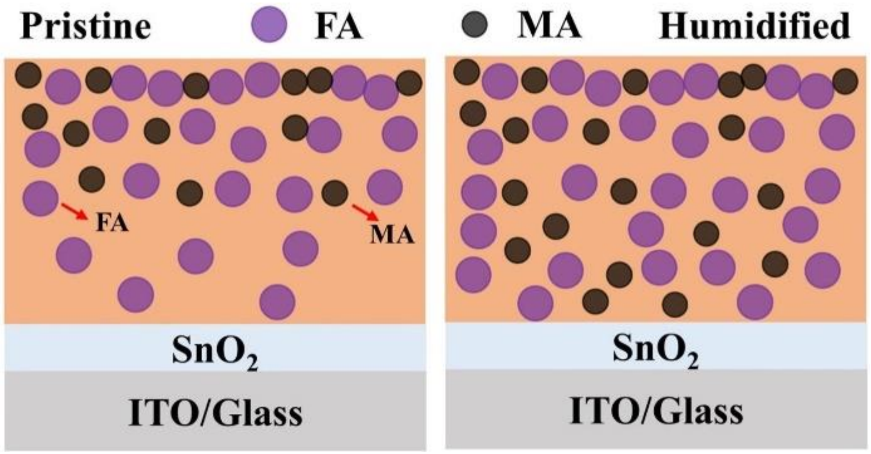

Supplementary Figure 6 | Vertical distribution of FA and MA in the intermediate perovskite films. Schematic diagram of the distribution of the FA and MA in the as-prepared wet intermediate perovskite film w/o (pristine) and with (humidified) moisture treatment. According to the results of depth XPS (Fig. 2 b and c), it can be speculated that moisture can facilitate the movement of MA to the bottom of the humidified intermediate perovskite film, achieving a more homogeneous phase distribution from the top to the bottom.

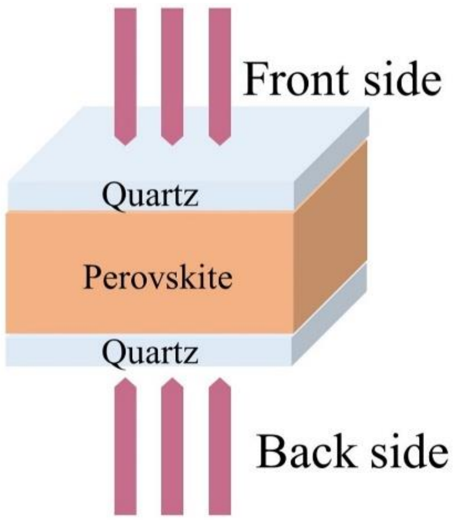

Supplementary Figure 7 | Illustration of the incident light of the PL measurements. Schematic diagram of measuring the PL spectra with different incident light paths. The upper quartz is coated on perovskite films to eliminate the possible influence of quartz substrates on the incident light intensity from different sides.

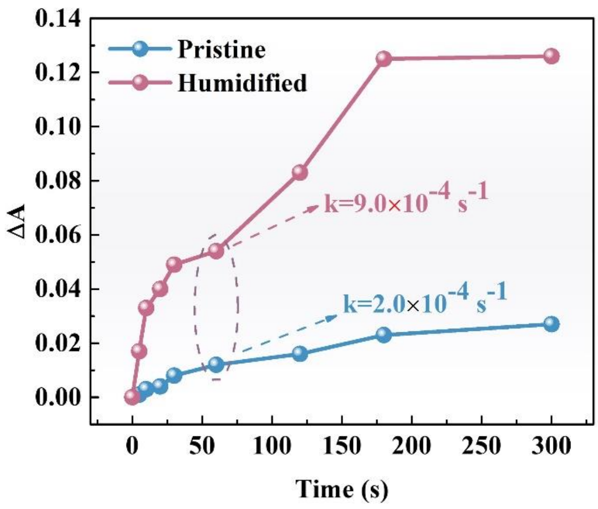

Supplementary Figure 8 | Effect of moisture on the evolution of absorbance as a function of time. Absorbance changes of the as-prepared wet intermediate perovskite film w/o (pristine) and with (humidified) moisture treatment under different times. ΔA is the absorbance difference of the perovskite film at 780 nm under different times with the initial value. It can be seen that the ΔA of humidified perovskite film increased more rapidly than the pristine, indicating the role of moisture treatment in accelerating the formation of perovskite crystallization sites.

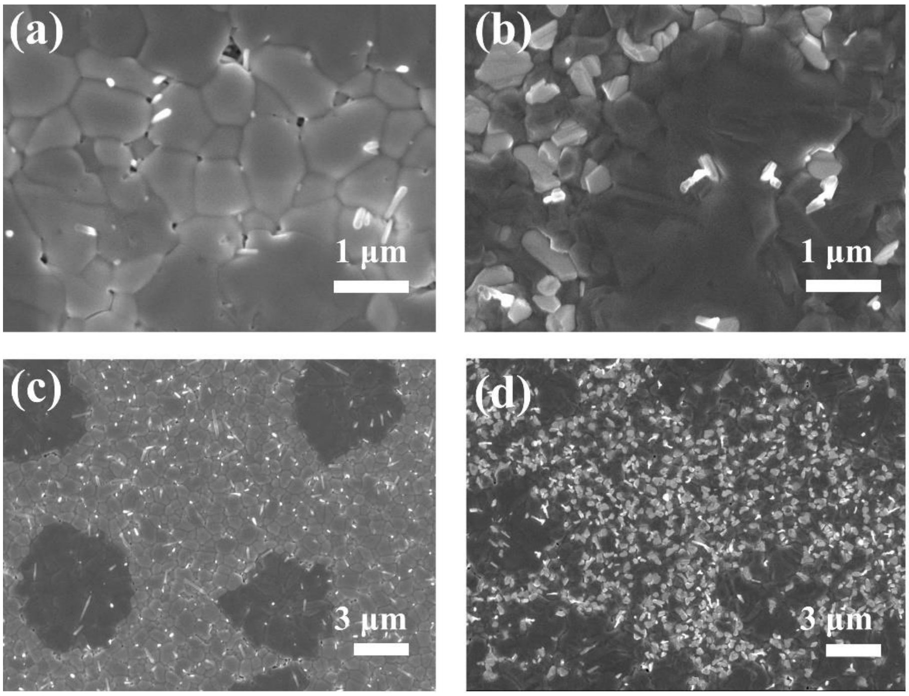

Supplementary Figure 9 | Top-view SEM images of perovskite films. (a, c) the over humidified films and (b, d) the subsequently annealed films.

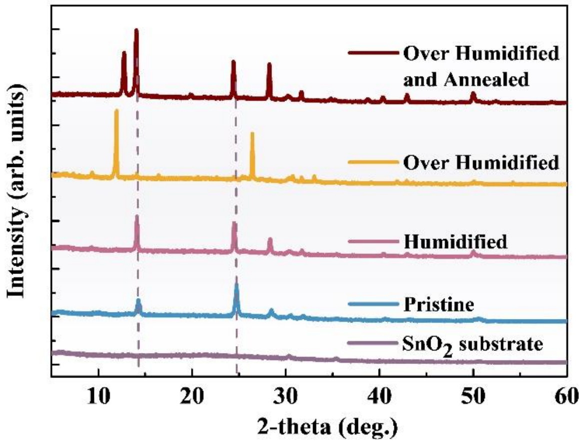

Supplementary Figure 10 | Effect of moisture on the evolution of XRD patterns of the perovskite films. The XRD patterns were measured on the same intermediate perovskite film at different moisture-treatment stages, including that, 1) Pristine, 2) Humidified, 3) Over Humidified, and 4) Over humidified and annealed.

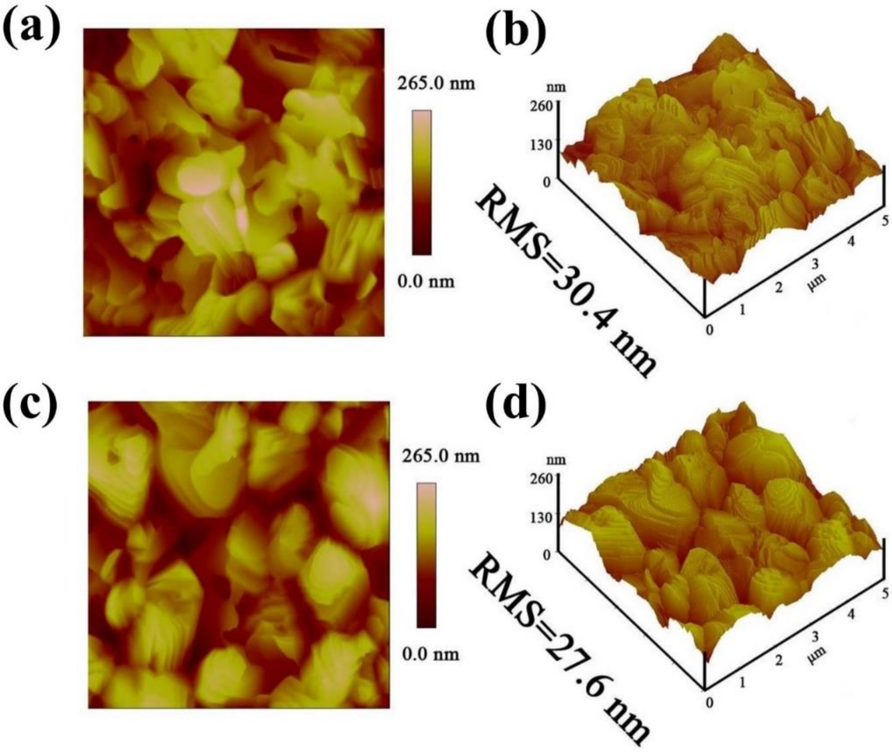

Supplementary Figure 11 | AFM measurements of perovskite films. (a, b) the control and (c, d) the target perovskite films (annealed). It can be seen that the target perovskite film contains larger crystal grains with lower roughness.

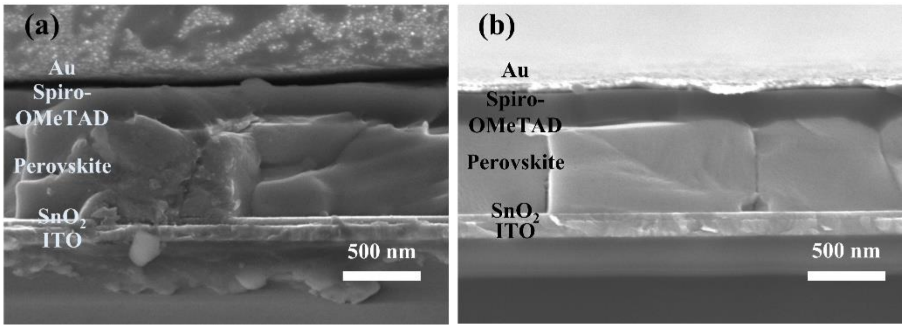

Supplementary Figure 12 | Cross-sectional SEM images of PSCs. Full solar cell device based on (a) the control and (b) the target perovskite film (annealed). The device structure is ITO/SnO_2/Perovskite/Spiro-OMeTAD/Au.

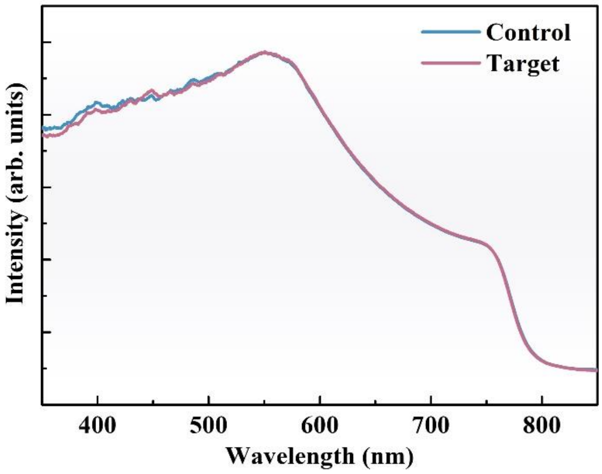

Supplementary Figure 13 | UV-vis absorption spectra of perovskite films. The control and the target perovskite films (annealed) show similar absorption intensity in the range of 350 to 850 nm.

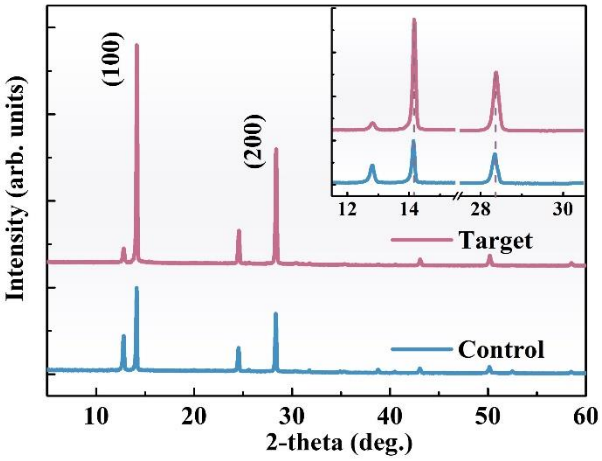

Supplementary Figure 14 | XRD patterns of the annealed perovskite films. The control and the target perovskite films (annealed) present the characteristic peaks of the perovskite phase.

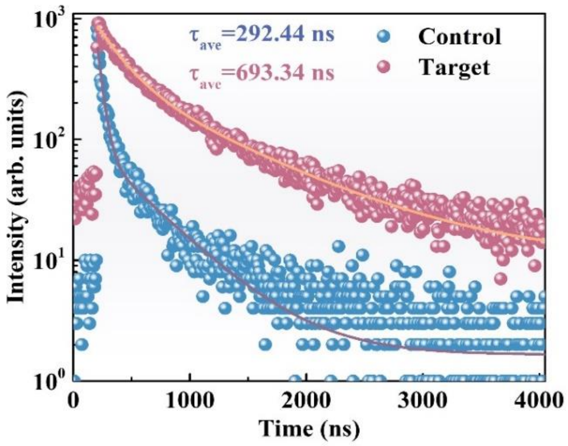

Supplementary Figure 15 | Time-resolved photoluminescence (TRPL) of perovskite films. The control and the target perovskite films (annealed) were deposited on the glass substrates without ITO and the charge transport layer.

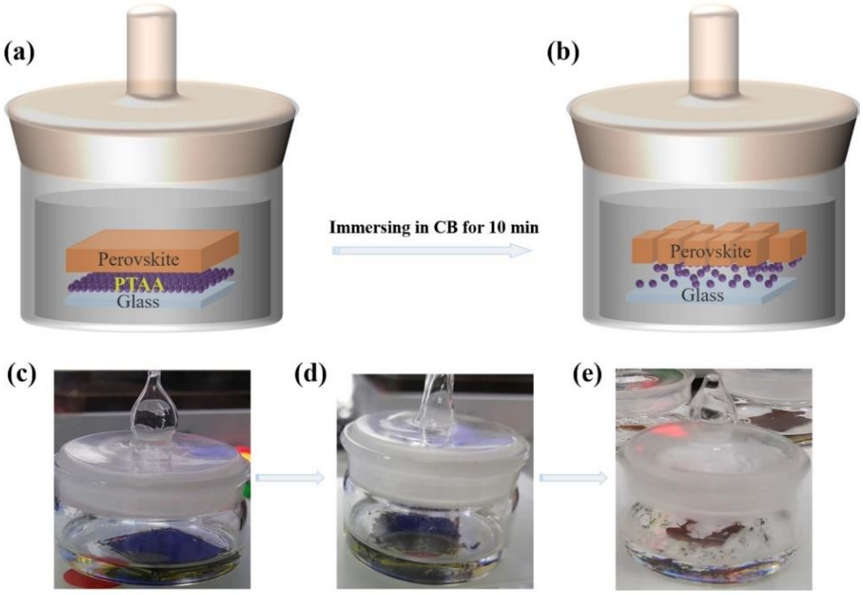

Supplementary Figure 16 | Schematic and photographs of the process of peeling and collecting the wet intermediate perovskite films for thermogravimetric analysis. (a) Immersing perovskite films deposited on glass/ITO/PTAA substrates in chlorobenzene (CB). (b) After the bottom PTAA film was dissolved, the upper perovskite films can be peeled off. (c)-(e) The photographs of the experiment process of peeling off perovskite films.

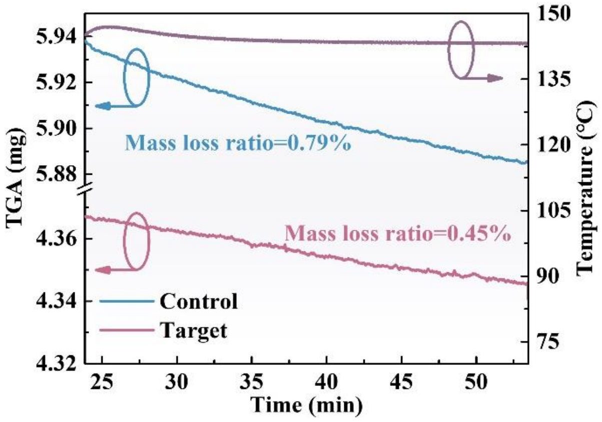

Supplementary Figure 17 | Thermal stability analysis of perovskite films. Thermal stability of the control and the target film as a function of time measured by TGA at 150 ℃ in N_2 (Simulating the annealing process of the intermediate perovskite film).

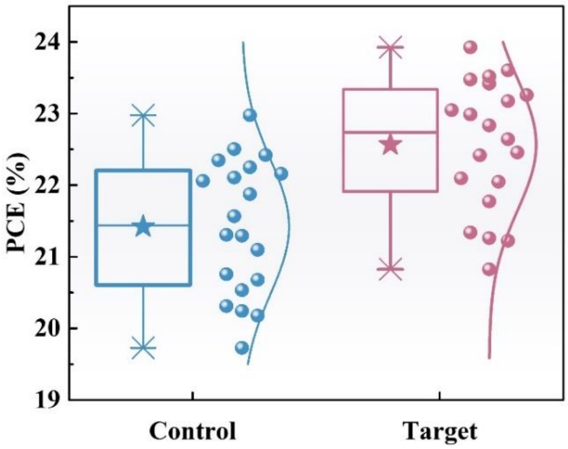

Supplementary Figure 18 | Statistical PCE of perovskite solar cells. Twenty control devices and twenty target devices are shown in the box chart.

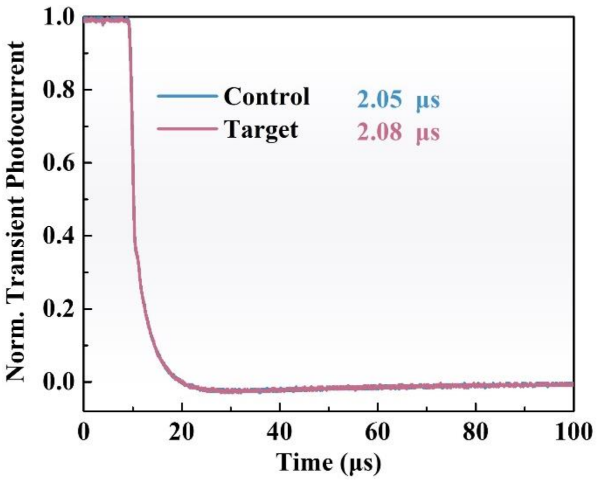

Supplementary Figure 19 | Transient photo-current decay curves of perovskite solar cells. The control and target device were based on the pristine perovskite film (without moisture treatment) and the humidified perovskite film (with moisture treatment), respectively. ("Norm." is abbreviated from the "normalized".)

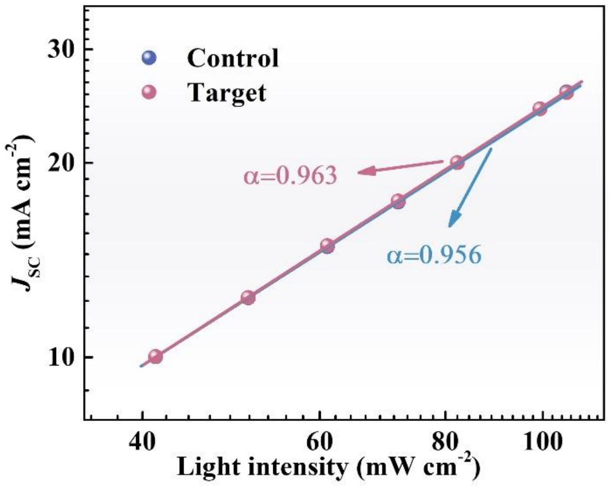

Supplementary Figure 20 | Evolution of J_{SC} as a function of light intensity. The dependency of J_{SC} on light intensity for the control and target devices.

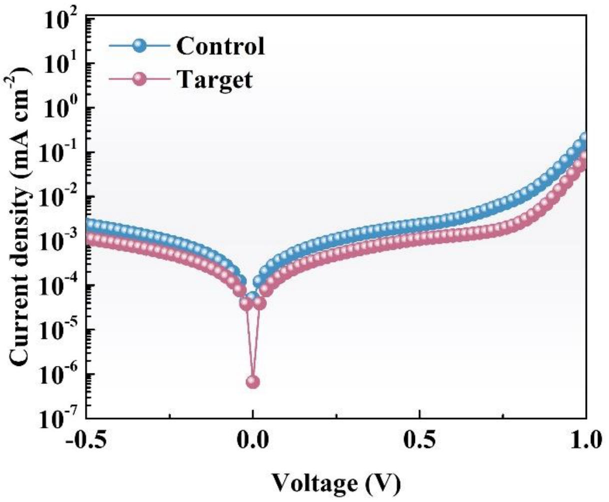

Supplementary Figure 21 | Dark *J*-*V* curves of perovskite solar cells. Compared with the control device, a lower leakage current for the target device is observed.

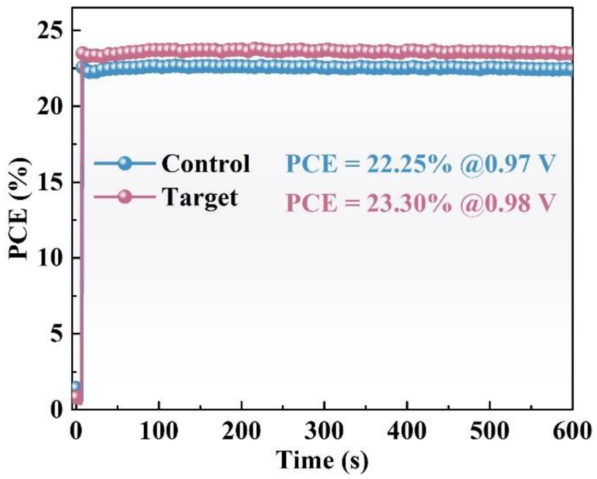

Supplementary Figure 22 | Stabilized power output of perovskite solar cells. The steady-state current density was measured at the maximum power point for the control and target devices.

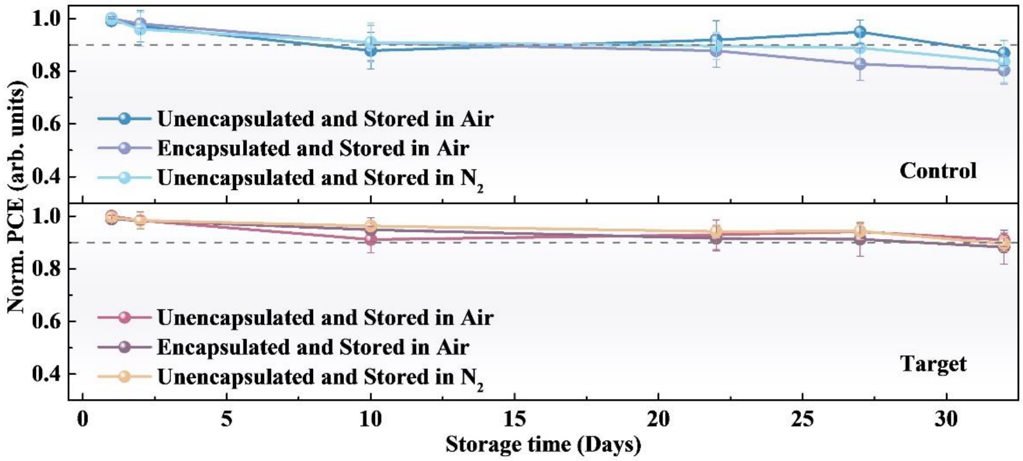

Supplementary Figure 23 | Shelf stability of perovskite solar cells. Shelf stability of the control and target devices under different storage conditions: 1) Unencapsulated and stored in the air; 2) Encapsulated and stored in the air; 3) Unencapsulated and stored in N_2. All the error bars represent the standard deviation for 10 devices, except for that of the control device with encapsulation representing 8 devices. 'Normalized' is denoted as 'Norm.'

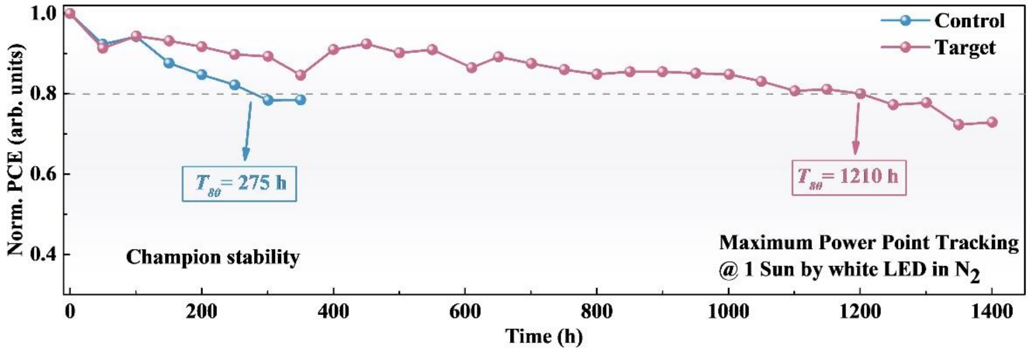

Supplementary Figure 24 | The champion operational stability of control and target devices. The data were tested under the maximum power point at 1-sun illumination in an N_2-filled glove box. 'Normalized' is denoted as 'Norm.'

Supplementary Note 1. Investigation of the composition of the perovskite film

We adopted chemical element analysis, and UV-Vis spectra to investigate the composition of perovskite. First, the ratio of Cs^+ in the A site is determined to be 0.05 as the Cs/Pb ratio is fixed as 0.05 in the precursor solution (CsI and PbI_2 dissolved in DMF/DMSO). The FA and MA ratios can't directly be determined by the precursor in the organic salts solution because they compete with each other to enter the PbI_2 film. Therefore, we collected perovskite powder that was peeled from the perovskite films to investigate the chemical element composition (Vario EL Cube). The obtained C/N ratio was determined to be about 0.48. So, the ratio of FA and MA was determined to be 0.75 and 0.20, respectively. Next, we fabricated perovskite films with varied molar ratios of MABr/MAI and fixed Cs, FA, and MA ratios in the one-step method. Note that the composition of the samples fabricated by the one-step method can be directly determined by the precursor. Finally, the I/Br ratio is determined by comparing the UV-vis absorption spectra of the target perovskite film with the known $Cs_{0.05}FA_{0.75}MA_{0.20}Pb(I_{1-x}Br_x)_3$ perovskite and x is determined to be 0.04. It is reported that Cl escapes after annealing and there is no residual Cl in the final perovskite film[1,2]. Finally, the perovskite composition in this work can be determined to be $(Cs_{0.05}FA_{0.75}MA_{0.20})Pb(I_{0.96}Br_{0.04})_3$.

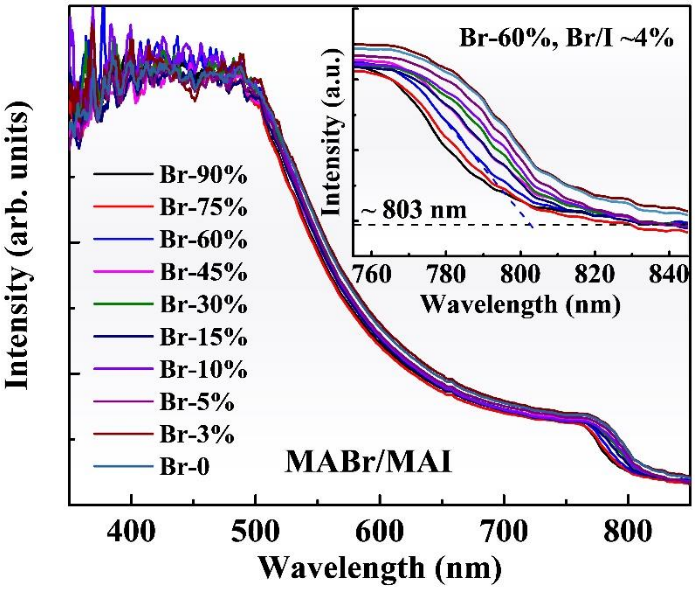

Supplementary Figure 25 | Analysis of the perovskite composition. UV-Vis spectra of the perovskite films with the composition of $(Cs_{0.05}FA_{0.75}MA_{0.20})Pb(I_{1-x}Br_{x})_3$. The bromide percentage in the legend represents different molar ratios between MABr and MAI in the precursor of the one-step method.

Supplementary Table 1 | Comparation of the roles of H_2O in fabricating perovskite with different compositions.

Serial number	Perovskite	Processing method/Roles of H_2O	Research method (Samples/ Characterizations)	PCE (%)	Stability	Year/Ref.
**1.**	$MAPbI_{3-x}Cl_x$	**Precursors** (2 vol% in DMF)/ Lowering the boiling point of the solvent to control the perovskite growth.	Annealed perovskite films/ UV-vis absorption spectroscopy and SEM, *etc.*	16.06	T_{90} >150 h; Stored in ambient conditions; Unencapsulated	2015/ Gong, X. et al.[3]
**2.**	$MAPbI_{3-x}Cl_x$	**Annealing in the ai**r/ Ensure fewer nucleation canters leading to large grains.	Annealed perovskite films/ PL, Absorption and SEM, *etc.*	16.7	-	2015/ Pathak, S. et al.[4]
**3.**	$MAPbI_{3-x}Cl_x$	**Annealing in the ai**r/ Provide an aqueous environment, enhance the ions diffusion length	Annealed perovskite films/ SEM, PL, TRPL, *etc.*	17.1	-	2014/ You, J. et al.[5]
**4.**	$MAPbI_{3-x}Cl_x$	**Annealing** (0-50% RH)/ Accelerates the crystal formation and enhances the texture orientation of the perovskite	The whole annealing process of perovskite/ *in situ* GIWAXS, UV-vis, SEM, *etc.*	19.04	T_{80} >1100 h (Under 1-sun illumination); T_{80} >1200 h (Thermal ageing at 65 ℃ in N_2); T_{80} >7800 h (Unencapsulated devices stored in the air, 298 K, 50% RH)	2021/ Huang, H.-H. et al.[6]

Supplementary Table 1 (Continued)

Serial number	Perovskite	Processing method/Roles of H_2O	Research method (Samples/ Characterizations)	PCE (%)	Stability	Year/Ref.
**5.**	$MAPbI_3$	**Precursors** (2 wt% H_2O in DMF)/ Make a homogenous precursor solution	Annealed perovskite films/ SEM and XRD, *etc.*	18	T_{90}>40 days; Stored in N_2; Unencapsulated	2015/ Wu, C.-G. et al.[7]
**6.**	$MAPbI_3$	**Precursors** (5 vol% H_2O in IPA) /Facilitate the reaction between the PbI_2 and MAI	Annealed perovskite films/ UV-vis, XRD and AFM, *etc.*	12.42	-	2016/ Adhikari, N. et al.[8]
**7.**	$MAPbI_3$	**Spin coating** (Expose TiO_2 substrates to the air)/ Pre-wetting the substrates forming porous PbI_2 films	The PbI_2 films and annealed perovskite films/ XRD, UV-vis and SEM, *etc.*	16.63	-	2015/ Lee, Y. H. et al.[9]
**8.**	$MAPbI_3$	**Spin coating** (Expose PbI_2 films to moisture, 1-60% RH)/ Fuse adjacent particles together forming large grains	The PbI_2 films and annealed perovskite films/ UV-vis, XRD and SEM, *etc.*	12.2	-	2016/ Gangishetty, M. K.[10]
**9.**	$MAPbI_3$	**Annealing** (Annealing in 30, 50, 75% RH)/ Deteriorated the morphology but improved the crystallinity	Annealed perovskite films/ XRD, SEM and AFM, *etc.*	14.28	-	2017/ Yin, X. et al.[11]

Supplementary Table 1 (Continued)

Serial number	Perovskite	Processing method/Roles of H_2O	Research method (Samples/ Characterizations)	PCE (%)	Stability	Year/Ref.
**10.**	$MAPbI_3$	**Annealing**<br>(Annealing in ambient conditions with 30-45% RH)/<br>Facilitate the formation of PbI_2 to passivate the grain boundaries	Annealed perovskite films/ SMM and AFM, *etc.*	17	-	2017/ Berweger, S. et al.[12]
**11.**	$MAPbI_3$	**Spin-coating, Post-treatment**<br>(With different RH)/<br>Self-healing perovskite lattices	The annealing process/ in situ absorbance; Annealed perovskite films/ SEM, XRD, *etc.*	18.5	-	2015/ Eperon, G. E. et al.[13]
**12.**	$MAPbI_3$	**Post-treatment**<br>(Expose to air, ~80% RH) /Convert the as-obtained perovskite to monohydrate and dihydrate species	Annealed perovskite films/ XRD, AFM, SEM and Ellipsometry, *etc.*	12.90	-	2015/ Leguy, A. M. A. et al.[14]
**13.**	$MAPbI_3$	**Precursors, spin-coating, and annealing**<br>(5 wt% H_2O in DMF & 0.5 vol% H_2O in IPA, spin-coating, and annealing in ambient)/<br>Help MAI penetrate the thick PbI_2 forming a thick film	Annealed perovskite films/ SEM and GIWXRD, *etc.*	20.1	T_{80} =30 days (Storage in ambient, 30-35% RH); Encapsulated	2017/ Chiang, C.-H. et al.[15]

Supplementary Table 1 (Continued)

Serial number	Perovskite	Processing method/Roles of H_2O	Research method (Samples/ Characterizations)	PCE (%)	Stability	Year/Ref.
**14.**	$MAPbI_3$	**Device Storage** (Ambient air with 30-40% RH) /Leading the cell degradation	Annealed perovskite films/ SEM, GIXRD, and UV-vis, *etc.*; Perovskite devices/ Long-term stability	18.03	T_{80} >30 days, Stored in N_2; T_{60} >10 days; Stored in ambient with 30-40% RH; Encapsulated	2016/ Chiang, C.-H. & Wu, C.-G.[16]
**15.**	$CsPbI_{3-x}Br_x$	**Post-treatment** (Exposure to moisture with about 20-80% RH)/ Moisture-mediated the transition from high-T to low-T phase	The fabricated perovskite films/ XRD, Absorption and PL, *etc.*	7	Retain 85% of the peak PCE after 40 repeated cycles of transitions	2018/ Lin, J. et al.[17]
**16.**	FA_xMA_{1-x}-PbI_yBr_{3-y}	**Annealing** (30-60% RH)/ Solubilize the organic halides for healing the perovskite film	Annealed perovskite films/ SEM, AFM, XRD and CLSM, *etc.*	19.46	$T_{96.8}$ >1000 h (Storage in N_2); $T_{87.9}$ >96 h (Storage in Air with 40% RH); Unencapsulated	2019/ Zhou, X. et al.[18]
**17.**	$(FAPbI_3)_{0.97}(MAPbBr_3)_{0.03}$	**Annealing** (30-40% RH and 70-80% RH) /Water interacts with perovskite to heal the defects	Annealed perovskite films/ in situ GI-XRD, PL, SEM, *etc.*	22.2	-	2021/ Meng, K. et al.[19]

Supplementary Table 1 (Continued)

Serial number	Perovskite	Processing method/Roles of H_2O	Research method (Samples/ Characterizations)	PCE (%)	Stability	Year/Ref.
**18.**	$Cs_{0.1}FA_{0.9}Pb$-$(I_{0.83}Br_{0.17})_3$	**Precursors** Prepare precursor solution with water as solvent	Annealed perovskite films/ XRD, SEM, XPS, *etc.*	13.0	-	2018/ Sveinbjörnsson, K. et al.[20]
**19.**	$(BA)_2(MA_{0.8}$-$FA_{0.15}Cs_{0.05})_4$ Pb_5I_{16}	**Precursors** (0-4 vol% H_2O in precursor solution)/ Form $MAI{\cdot}H_2O$, modulate crystal orientation and phase distribution	Annealed perovskite films/ PL, Confocal PL, TA, GIWAXS and NMR, *etc.*	18.04	T_{85} > 500 h (Thermal ageing at 60 ℃)	2020/ Li, X. et al.[21]
**20.**	$Cs_{0.05}(FA_{0.83}$-$MA_{0.17})_{0.95}Pb($ $I_{0.83}Br_{0.17})_3$	**Precursors** (0-5 vol% H_2O in precursor solution)/ Hydrate water induces the recrystallization of perovskite films	Annealed perovskite films/ (XRD, Absorption, PL and SEM, *etc.*	19.4	T_{80} = 200 h (Storage in Air, 35-45% RH); Unencapsulated	2019/ Zhang, W. H. et al.[22]
**21.**	$Cs_{0.05}(FA_{0.83}$-$MA_{0.17})_{0.95}Pb($ $I_{0.83}Br_{0.17})_3$	**Post-treatment** (Exposure to moisture with 80% RH for 48 h) /Induce a crystallographic reorientation in the perovskite films	Annealed perovskite films/ GIWAXS, PL, Absorption, XRF and XBIC, *etc.*	18.0	A slow decrease in the PCE after a week	2020/ Hidalgo, J. et al.[23]

Supplementary Table 1 (Continued)

Serial number	Perovskite	Processing method/Roles of H_2O	Research method (Samples/ Characterizations)	PCE (%)	Stability	Year/Ref.
**22.**	$Cs_{0.05}(FA_{0.83}$-$MA_{0.17})_{0.95}Pb(I_{0.83}Br_{0.17})_3$	**Post-treatment** (Exposure to moisture, 70% RH)/ Moisture-based passivation and degradation for perovskite films	Annealed perovskite films/ in situ PL	-	-	2021/ Howard, J. M. et al.[24]
**23.**	$Cs_{0.05}(FA_{0.83}$-$MA_{0.17})_{0.95}Pb(I_{0.83}Br_{0.17})_3$	**Post-treatment** (Exposure to moisture, 85% RH)/ Moisture induces the decomposition of perovskite into PbI_2 or the non-equilibrium phase segregation	Annealed perovskite films/ in situ XRD, in situ TEM and NMR, *etc.*	-	-	2021/ Kazemi, M. A. A. et al.[25]
**24.**	$(Cs_{0.05}FA_{0.75}$-$MA_{0.20})Pb(I_{0.96}Br_{0.04})_3$	**Pre-humidified** (~35% RH)/ Enable a spatially homogeneous intermediate phase for the wet film	The protected wet perovskite films before annealing/ in situ UV-vis, in situ SEM, depth XPS, NMR and TGA, *etc.*	23.93	T_{80} > 850 h; Under 1-sun illumination; Unencapsulated	**This work**

Abbreviations in Table 3: (1) $MAPbI_3$ represents $CH_3NH_3PbI_3$; (2) The relative humidity (RH); (3) UV-vis absorption spectra (UV-vis); (4) Scanning electron microscopy (SEM); (5) X-ray diffraction (XRD); (6) Steady photoluminescence (PL); (7) Time-resolved photoluminescence (TRPL) (8) Grazing-incident wide-angle X-ray scattering (GIWAXS); (9) Atomic force microscopy (AFM); (10) Grazing incidence X-ray diffraction (GIXRD or GI-XRD); (11) Scanning microwave microscopy (SMM); (12) Confocal laser scanning microscopy (CLSM); (13) X-ray fluorescence (XRF); (14) X-ray beam-induced current (XBIC); (15) Transient absorption spectra (TA); (16) "-" represents "Not mentioned".

Supplementary Table 2 | Integrated area for the peak of H_2O and DMSO-d6 in NMR results. (To compare the semiquantitative content of water molecules in these three samples, we deducted the ratio value of the DMSO-d6 sample to gain the relative value of the absorbed water molecules in the pristine and humidified sample.)

Sample	H_2O	DMSO-d6	Relative Ratio (%)	Deducted the DMSO-d6 (%)
DMSO-d6	0.98	29.02	3.38	0
Pristine	1.18	23.20	5.09	1.71
Humidified	2.01	32.27	6.23	2.85

Supplementary Table 3 | Fitted parameters of the TRPL results.

Perovskite film	τ_1 (ns)	A_1	τ_2 (ns)	A_2	τ_{ave} (ns)
Control	46.59	515.82	463.85	74.31	292.44
Target	221.71	547.12	902.21	303.59	693.34

Supplementary Methods

Characterization of perovskite films

Time-resolved photoluminescence (TRPL) was studied by FLS920 (Edinburgh Instruments, Ltd.). Perovskite films were directly deposited on glass substrates to evaluate the lifetime of carriers. TRPL results were fitted following the bi-exponential decay equation:

$$I(t) = I_0 + A_1 \exp(-t/\tau_1) + A_2 \exp(-t/\tau_2) \quad (1)$$

The average lifetime (τ_{ave}) of the control perovskite film and the humidified perovskite film was calculated via the following equation:

$$\tau_{ave} = (A_1 \tau_1^2 + A_2 \tau_2^2)/(A_1 \tau_1 + A_2 \tau_2) \quad (2)$$

where A_1 and A_2 is the amplitude for the fast and slow decay component, respectively, and τ_1 and τ_2 are the time constant for the fast and slow decay components, respectively.[26]

Chemical element analysis was recorded by Vario EL Cube. The sample of perovskite powder was collected from many perovskite films.

Device characterizations

Space-charge-limited current (SCLC) analysis, electron-only devices were fabricated with the configuration of ITO/SnO_2/perovskite (control or target)/PCBM/Au, where ITO is indium tin oxide and PCBM is [6,6]-phenyl-C_{61}-butyric acid methyl ester. Subsequently, measuring their dark current density-voltage (J–V) curves can obtain the trap-filled limit voltage (V_{TFL}) and then calculated the corresponding total trap densities in perovskite film via the equation that：

$$n_{trap} = V_{TFL} \cdot \frac{2\varepsilon\varepsilon_0}{qL^2} \quad (3)$$

where q is the elementary charge, L is the thickness of perovskite film, ε is the relative permittivity, ε_0 is the vacuum permittivity, and V_{TFL} is the voltage of the kink-point in the *J*-*V* curve, respectively.[27]

Mott-Schottky analysis was achieved by taking the capacitance-voltage (*C*-*V*) curves under dark conditions, where the built-in potential (V_{bi}) followed the Mott-Schottky equation that：

$$\frac{1}{C^2} = \frac{2(V_{bi}-V)}{A^2 e\varepsilon\varepsilon_0 N} \quad (4)$$

where A, e ε, ε_0, and N are device area, the elementary charge, relative permittivity, vacuum permittivity, and carrier concentration, respectively.[28]

Thermal admittance spectra were measured at room temperature with the complete solar cell device. The frequency range was 100 Hz to 1 MHz. V_{bi} and W were extracted from the Mott-Schottky curves, and then the trap density of states (tDOS) of both control and target devices were calculated with the equation：

$$N_t = -\frac{V_{bi}}{qW}\frac{dC}{d\omega}\frac{\omega}{kT} \quad (5)$$

where C, ω, k, and T are the capacitance, applied angular frequency, the Boltzmann constant, and temperature, respectively.[29]

The dependency of V_{OC} and J_{SC} as a function of the incident light intensity was obtained by measuring the *J*–*V* curve under different light intensities. The ideality factor n of devices was calculated following the equation：

$$V_{OC} = \frac{nKT}{q ln(\frac{I_{SC}}{I_0}+1)} \quad (6)$$

where K, T, I_{SC}, I_0, and q are the Boltzmann factor, temperature, current density, saturated current density, and elementary charge, respectively.[30]

Supplementary References

1. Kim, M. *et al.* Methylammonium chloride induces intermediate phase stabilization for efficient perovskite solar cells. *Joule* **3**, 2179-2192 (2019).
2. Ye, F. *et al.* Roles of macl in sequentially deposited bromine-free perovskite absorbers for efficient solar cells. *Adv. Mater.* **33**, 2007126 (2021).
3. Gong, X. *et al.* Controllable perovskite crystallization by water additive for high-performance solar cells. *Adv. Funct. Mater.* **25**, 6671-6678 (2015).
4. Pathak, S. *et al.* Atmospheric influence upon crystallization and electronic disorder and its impact on the photophysical properties of organic-inorganic perovskite solar cells. *ACS Nano* **9**, 2311-2320 (2015).
5. You, J. *et al.* Moisture assisted perovskite film growth for high performance solar cells. *Appl. Phys. Lett.* **105**, 183902 (2014).
6. Huang, H.-H. *et al.* Mild water intake orients crystal formation imparting high tolerance on unencapsulated halide perovskite solar cells. *Cell Rep. Phys. Sci.* **2**, 100395 (2021).
7. Wu, C.-G. *et al.* High efficiency stable inverted perovskite solar cells without current hysteresis. *Energy Environ. Sci.* **8**, 2725-2733 (2015).
8. Adhikari, N. *et al.* Crystallization of a perovskite film for higher performance solar cells by controlling water concentration in methyl ammonium iodide precursor solution. *Nanoscale* **8**, 2693-2703 (2016).
9. Lee, Y. H. *et al.* Unraveling the reasons for efficiency loss in perovskite solar cells. *Adv. Funct. Mater.* **25**, 3925-3933 (2015).
10. Gangishetty, M. K., Scott, R. W. J. & Kelly, T. L. Effect of relative humidity on crystal growth, device performance and hysteresis in planar heterojunction perovskite solar cells. *Nanoscale* **8**, 6300-6307 (2016).

11. Yin, X. *et al.* Moisture annealing effect on $CH_3NH_3PbI_3$ films deposited by solvent engineering method. *Thin Solid Films* **636**, 664-670 (2017).

12. Berweger, S. *et al.* Electronic and morphological inhomogeneities in pristine and deteriorated perovskite photovoltaic films. *Nano Lett.* **17**, 1796-1801 (2017).

13. Eperon, G. E. *et al.* The importance of moisture in hybrid lead halide perovskite thin film fabrication. *ACS Nano* **9**, 9380-9393 (2015).

14. Leguy, A. M. A. *et al.* Reversible hydration of $CH_3NH_3PbI_3$ in films, single crystals, and solar cells. *Chem. Mater.* **27**, 3397-3407 (2015).

15. Chiang, C.-H., Nazeeruddin, M. K., Grätzel, M. & Wu, C.-G. The synergistic effect of H_2O and dmf towards stable and 20% efficiency inverted perovskite solar cells. *Energy Environ. Sci.* **10**, 808-817 (2017).

16. Chiang, C.-H. & Wu, C.-G. Film grain-size related long-term stability of inverted perovskite solar cells. *ChemSusChem* **9**, 2666-2672 (2016).

17. Lin, J. *et al.* Thermochromic halide perovskite solar cells. *Nat. Mater.* **17**, 261-267 (2018).

18. Zhou, X. *et al.* Role of moisture in the preparation of efficient planar perovskite solar cells. *ACS Sustainable Chem. Eng.* **7**, 17691-17696 (2019).

19. Meng, K. *et al.* Humidity-induced defect-healing of formamidinium-based perovskite films. *Small* **17**, 2104165 (2021).

20. Sveinbjörnsson, K. *et al.* Preparation of mixed-ion and inorganic perovskite films using water and isopropanol as solvents for solar cell applications. *Sustain. Energ. Fuels* **2**, 606-615 (2018).

21. Li, X. *et al.* Water-assisted crystal growth in quasi-2D perovskites with enhanced charge transport and photovoltaic performance. *Adv. Energy Mater.* **10**, 2001832 (2020).

22. Zhang, W. H., Xiong, J., Loi, J. H. & Daoud, W. A. Mechanism of water effect on enhancing the photovoltaic performance of triple-cation hybrid perovskite solar cells. *ACS Appl. Mater. Inter.* **11**, 12699-12708 (2019).

23. Hidalgo, J. *et al.* Moisture-induced crystallographic reorientations and effects on charge carrier extraction in metal halide perovskite solar cells. *ACS Energy Lett.* **5**, 3526-3534 (2020).

24. Howard, J. M. *et al.* Water-induced and wavelength-dependent light absorption and emission dynamics in triple-cation halide perovskites. *Adv. Opt. Mater.* **9**, 2100710 (2021).

25. Kazemi, M. A. A. *et al.* Moisture-induced non-equilibrium phase segregation in triple cation mixed halide perovskite monitored by in situ characterization techniques and solid-state nmr. *Energy Environ. Mater.* **0**, 1–10 (2021).

26. Zhu, J. *et al.* Formamidine disulfide oxidant as a localised electron scavenger for >20% perovskite solar cell modules. *Energy Environ. Sci.* **14**, 4903-4914 (2021).

27. Liu, T. *et al.* Spacer engineering using aromatic formamidinium in 2D/3D hybrid perovskites for highly efficient solar cells. *ACS Nano* **15**, 7811-7820 (2021).

28. Zhang, C. C. *et al.* Electric-field assisted perovskite crystallization for high-performance solar cells. *J Mater. Chem. A* **6**, 1161-1170 (2018).

29. Liu, C. *et al.* α-$CsPbI_3$ bilayers via one-step deposition for efficient and stable all-inorganic perovskite solar cells. *Adv. Mater.* **32**, e2002632 (2020).

30. Singh, T. & Miyasaka, T. Stabilizing the efficiency beyond 20% with a mixed cation perovskite solar cell fabricated in ambient air under controlled humidity. *Adv. Energy Mater.* **8**, 1700677 (2018).
